# Paralytic shellfish toxin content is related to genomic *sxtA4* copy number in *Alexandrium minutum* strains

**DOI:** 10.3389/fmicb.2015.00404

**Published:** 2015-05-01

**Authors:** Anke Stüken, Pilar Riobó, José Franco, Kjetill S. Jakobsen, Laure Guillou, Rosa I. Figueroa

**Affiliations:** ^1^Department of Biosciences, University of OsloOslo, Norway; ^2^U.A. Microalgas Nocivas (Consejo Superior de Investigaciones Científicas – Instituto Español de Oceanografía), Instituto de Investigaciones MarinasVigo, Spain; ^3^Centre for Ecological and Evolutionary Synthesis, Department of Biosciences, University of OsloOslo, Norway; ^4^Laboratoire Adaptation et Diversité en Milieu Marin, CNRS, UMR 7144Roscoff, France; ^5^Sorbonne Universités – Université Pierre et Marie Curie, UMR 7144Roscoff, France; ^6^Aquatic Ecology, Lund UniversityLund, Sweden; ^7^U.A. Microalgas Nocivas (Consejo Superior de Investigaciones Científicas – Instituto Español de Oceanografía), Instituto Español de OceanografíaVigo, Spain

**Keywords:** Dinoflagellate, *Alexandrium*, saxitoxin (STX), paralytic shellfish toxin (PST), *sxtA*, gene dosage, copy number variation, genome size

## Abstract

Dinoflagellates are microscopic aquatic eukaryotes with huge genomes and an unusual cell regulation. For example, most genes are present in numerous copies and all copies seem to be obligatorily transcribed. The consequence of the gene copy number (CPN) for final protein synthesis is, however, not clear. One such gene is *sxtA*, the starting gene of paralytic shellfish toxin (PST) synthesis. PSTs are small neurotoxic compounds that can accumulate in the food chain and cause serious poisoning incidences when ingested. They are produced by dinoflagellates of the genera *Alexandrium*, *Gymnodium*, and *Pyrodinium*. Here we investigated if the genomic CPN of *sxtA4* is related to PST content in *Alexandrium minutum* cells. *SxtA4* is the 4th domain of the *sxtA* gene and its presence is essential for PST synthesis in dinoflagellates. We used PST and genome size measurements as well as quantitative PCR to analyze *sxtA4* CPN and toxin content in 15 *A. minutum* strains. Our results show a strong positive correlation between the *sxtA4* CPN and the total amount of PST produced in actively growing *A. minutum* cells. This correlation was independent of the toxin profile produced, as long as the strain contained the genomic domains *sxtA1* and *sxtA4*.

## Introduction

“From the beginning, the problem of mussel poisoning appeared to be complex,” Dr. R. Stohler, 1937 (Sommer et al., 1937). Over 60 years later, despite many great advances in research on origin and diversity of mussel toxins, the phenomenon of paralytic shellfish poisoning (PSP) is still not fully explained. Stohler was co-author on a series of papers that summarized the knowledge on PSP at the time. Among the main breakthroughs was the identification of the dinoflagellate *Alexandrium catenella* (Whedon and Kofoid) Balech as the plankton species responsible for PSP outbreaks along the Pacific coast of North America. They showed that the mussels became toxic when they fed on *A. catenella* and gradually lost their toxicity after the mussels stopped feeding on it. Sommer et al. (1937) also demonstrated that the paralytic shellfish toxins (PSTs) were present in plankton samples containing *A. catenella*, that the PST content in the dinoflagellates varied and that it was likely to consist of more than one active substance.

Paralytic shellfish toxins are a group of small neurotoxic alkaloids that are among the most potent natural toxins known. To date, 57 isoforms of saxitoxin, the parent compound of PSTs, have been described (reviewed in Wiese et al., 2010). More PST-producing plankton species have also been identified. These include additional *Alexandrium* species, and the two dinoflagellates *Gymnodinium catenatum* Graham and *Pyrodinium bahamense* Plate, but also several species of freshwater cyanobacteria.

On a worldwide basis, *Alexandrium* species are the most abundant and widespread (Anderson et al., 2012) and much research has focused on identifying factors that influence PST synthesis in this genus (recent review: Anderson et al., 2012). About one third of the 31 taxonomically accepted *Alexandrium* species today have been reported to produce PSTs (Anderson et al., 2012; Guiry and Guiry, 2013). The mix of PST isoforms produced, i.e., the PST profile, appears to be fixed in each strain and is thought to be inherited in Mendelian fashion (Sako et al., 1992) but can vary between strains of the same species. The total amount of PSTs and the relative proportions of the PST isoforms produced, however, can vary in each strain in response to a range of biotic and abiotic factors. These include for example nutrient limitations (Boczar et al., 1988; Anderson et al., 1990; John and Flynn, 2000), intracellular arginine concentration (Anderson et al., 1990; John and Flynn, 2000), temperature (Anderson et al., 1990), and grazer presence (Bergkvist et al., 2008). In addition, strains that do not produce any detectable amounts of PSTs have also been reported to occur within otherwise PST-producing *Alexandrium* species.

Despite these advances, it is still not known how PST synthesis is regulated at a cellular level in dinoflagellates. This gap of knowledge is most likely due to the unusual genome organization of dinoflagellates. For one dinoflagellate genomes are huge. Haploid genome size measurements range from 1.5 to >225 pg cell per cell (Veldhuis et al., 1997; LaJeunesse et al., 2005) and thus correspond to 0.5 to >70 times the human haploid genome. The biggest part of dinoflagellate genomes is made up of simple and complex repeats (Allen et al., 1975; Davies et al., 1988; McEwan et al., 2008; Jaeckisch et al., 2011) and only about 0.2–1.8% of sequence code for protein coding genes (McEwan et al., 2008; Hou and Lin, 2009; Jaeckisch et al., 2011). These genes frequently occur in multiple copies and are often arranged in tandem arrays (Le et al., 1997; Li and Hastings, 1998; Bachvaroff and Place, 2008; Shoguchi et al., 2013), but single-copy-genes may also exist (Bachvaroff and Place, 2008). The different copies of multi-copy genes are often not identical (Lee et al., 1993; Machabée et al., 1994), and it appears as if all gene copies are constantly expressed (Machabée et al., 1994). Further, recent studies using whole transcriptome sequencing technology (Moustafa et al., 2010; Yang et al., 2010) or microarray analyses (Yang et al., 2011) suggest that only 0.35–27% of dinoflagellate genes are transcriptionally regulated.

The copy numbers (CPNs) of different genes within one species vary widely. For example, the dinoflagellate *Lingulodinium polyedra* (Stein) Dodge has been reported to contain ∼30 copies of a protein kinase gene (Salois and Morse, 1997), 146 copies of the luciferase gene (Liu and Hastings, 2005), ∼1,000 copies of the Luciferin-binding Protein genes (Lee et al., 1993) and ∼5,000 copies of the mitotic cyclin gene (Bertomeu and Morse, 2004). The importance of these high gene CPNs for the cellular biology of dinoflagellates is not clear. However, it has been suggested that they may be related to the amount of protein that can be synthesized by a dinoflagellate cell (Lee et al., 1993, 2014; Moustafa et al., 2010).

Recently, two research groups have identified transcripts and transcript fragments that are putatively involved in PST synthesis in dinoflagellates (Stüken et al., 2011; Hackett et al., 2013; Orr et al., 2013). Both groups have independently identified transcripts that are related to *sxtA* (Stüken et al., 2011; Hackett et al., 2013) the putative starting gene of PST synthesis in cyanobacteria (Kellmann et al., 2008). The cyanobacterial *sxtA* gene contains four catalytic domains, *sxtA1* to *sxtA4* (Kellmann et al., 2008) and dinoflagellate transcripts containing either *sxtA1–A3* or all four catalytic domains have been characterized in detail (Stüken et al., 2011). The organization of the four domains within the dinoflagellate genome, however, is unresolved: so far, only genomic fragments of *sxtA1* and *sxtA4* could be amplified, not the entire gene (Stüken et al., 2011). However, evidence is accumulating that PST-producing dinoflagellates contain genomic copies of *sxtA1* and *sxtA4*, whereas one or both of these domains are often not detected in dinoflagellates that do not produce PSTs (Murray et al., 2011b, 2012; Stüken et al., 2011; Orr et al., 2013; Suikkanen et al., 2013). In addition it has been shown that at least *sxtA4* occurs in multiple, non-identical genomic copies (Stüken et al., 2011; Wiese et al., 2014). These different copies are most likely constitutivly transcribed: divergent *sxtA1* and *sxtA4* transcript families have been detected in *Alexandrium* transcriptomes (Stüken et al., 2011). Further, specific *sxtA4* transcript analyses of *A. catenella* strains did not reveal any changes in transcript levels throughout the growth cycle, despite significant changes in PST production rates (Wiese et al., 2014).

The aim of this study was to investigate if there is a relationship between the genomic CPN of *sxtA4* and PST content in dinoflagellate cells. We focused on *A. minutum,* a globally distributed *Alexandrium* species that contains PST-producing and non-producing strains. We measured genome size and PST content and estimated the genomic *sxtA4* CPN in all strains using quantitative PCR methods.

## Materials and Methods

### Strains and Culture Conditions

The *A. minutum* strains studied are listed in **Table 1**. All strains were grown in L1 medium (Guillard and Hargraves, 1993) without added silica, at 30 PSU salinity, 16°C, a 12:12 h light–dark photoperiod and a photon irradiance of 90–100 μmol photons m^2^ s^-1^. Strains were xenic.

**Table 1:** Overview over the *Alexandrium minutum* strains used in this study, their genome size, genomic *sxtA4* CPNs, and average total PST content.

Strain	Isolation		Genome		*sxtA4*		Total PST		Toxin group
(Synonym)	Location	Year	Size [pg]	SD	CPN cell^-1^	SD	fmol cell^-1^	SD	^∗^
AL1V (CCMP113)	Ria de Vigo, Spain	1987	22.5	3.8	4.3	1.9	0.442	0.000	1
VGO722	Cambrils, Catalonia, Spain	2003	23.3	1.0	2.2	0.5	2.269	0.292	1
AMP13	Palma de Mallorca, Spain	1995	24.4	2.3	2.3	0.5	6.200	4.111	1
AL10C	Estartit, Catalonia, Spain	2002	24.5	2.2	2.4	0.9	0.115	0.026	1
VGO942	Adriatic Sea, Italy	2008	24.9	1.5	1.5	1.0	0.681	0.731	1
AL4V	Ria de Vigo, Spain	2000	25.2	2.6	7.0	5.4	2.358	0.360	1
Min3	Arenys, Catalonia, Spain	2002	25.2	2.2	2.0	1.1	0.650	0.439	1
VGO577	Girona, Catalonia, Spain	2002	25.7	3.2	6.4	0.5	6.574	4.336	1
AMP4	Palma de Mallorca, Spain	1995	26.2	3.0	4.0	2.2	8.446	2.762	1
VGO874	Boughrara, Tunesia	2006	29.0	3.4	2.8	0.2	0.531	0.181	1
RCC3227	Rance river, Brittany, France	2011	25.4	1.7	10.8	3.3	3.303	4.689	2
RCC3337	Penzé river, Brittany, France	2011	26.3	2.4	5.8	2.2	1.850	1.108	2
VGO650	Brittany, France	2003	26.5	1.7	6.3	0.7	15.915	3.343	2
VGO651	Brittany, France	2003	26.9	3.5	7.7	1.2	17.773	8.888	2
VGO663	Sardina, Italy	2003	29.6	2.6	N.D.		N.D.		3

SD, standard deviation; CPN, copy number per genome; N.D, not detected; ^∗^group 1, mainly GTX1/4 and GTX2/3; group 2, mainly C1/2, GTX2/3; group 3, no PST detected.

### Genome Size Measurements

Exponentially growing cultures (6,000–10,000 cells mL^-1^) were incubated 48 h in darkness to induce synchronization of cell division in *Alexandrium* (Taroncher-Oldenburg et al., 1997; Figueroa et al., 2010). Fifty milliliter of culture were filtered through a 5.0 μm isopore membrane filter (Millipore, Ireland), fixed with 1% paraformaldehyde for 10 min, and washed in PBS (pH 7, Sigma–Aldrich, St. Louis, MO, USA; 1200 g × 10 min). The pellet was re-suspended in 2 mL of cold methanol and stored for at least 12 h at 4°C to allow chlorophyll extraction. The cells were then washed twice in PBS and the pellet was re-suspended in a staining solution (PBS, 0.1 mg propidium iodide mL^-1^ and 2 μg RNaseA mL^-1^) for at least 2 h before analysis. A Beckman FC500 bench machine with a laser emitting at 488 nm was used. Four replicate samples were run at low speed (∼18 μL min^-1^) and data were acquired in linear and log modes until at least 1000 events had been recorded. As DNA standard, 10 μl of a triploid trout solution (7.8 pg nucleus^-1^, Biosure, USA) was added to each sample. Fluorescence emission of propidium iodide was detected at 620 nm. The software FlowJo 7.6 (Tree Star, Inc., USA) was used to compute peak numbers, coefficients of variation (CVs), and peak ratios for the DNA fluorescence distributions in a population. Runs with CVs above 10 were discarded from analyses.

### PST Measurements

The cultures grown for toxin analyses are listed in **Table 1**. They were grown in batch mode in 500 mL Erlenmeyer flasks containing 250 mL of L1 medium (see Strains and Culture Conditions for details). The cultures were initiated at a density of 500 cells mL^-1^ provided by the inoculation of each flask with acclimated exponentially growing cells. For growth monitoring 5 mL samples were collected every 3 days, fixed with Lugol’s solution and cell counts in Sedgewick–Rafter slides. For toxin analyses, three 50 ml aliquots were harvested in actively growing cultures using two different inocula. Cultures were filtered using 1.4 μm GF/C glass fiber filters (25 mm 𝜃). Toxins in cells were extracted with 1000–2500 μL acetic acid 0.05 M depending on cell concentration.

The PSP toxins were separated using the method proposed by Rourke et al. (2008) Standard solutions of GTX4,1, dcGTX2,3, GTX2,3, STX, neoSTX, and dcSTX were purchased from the Institute for Marine Bioscience, National Research Council, Certified Reference Material Program (NRC–CRM), Halifax, NS, Canada. Toxin concentrations were determined by comparing the peak area for each toxin with that of the standard.

### DNA Isolation and Quantification

Genomic DNA was isolated using the following Cetyl Trimethyl Ammonium Bromide (CTAB) protocol: *Alexandrium* cultures were harvested through centrifugation and pellets were stored at -80°C. To isolate DNA, 700 μl CTAB buffer containing 4 (v/v) 2-Mercapthoethanol and 0.1 mg ml^-1^ Proteinase K was added to each thawed pellet. Samples were vortexed, then incubated for 1 h at 65°C during which they were inverted every 10 min. Samples were let to cool, 700 μl chloroform: isoamyl alcohol (24:1) was added, samples were mixed by inversion and then shaken horizontally for 20 min. Afterward, samples were centrifuged at 4°C, 15,700 × *g* for 20 min, the upper aqueous phase was transferred to a fresh tube and the chloroform: isoamyl alcohol step was repeated on the transferred extract. Then 1 μl RNAse A (10 mg ml^-1^) was added and samples were incubated for 30 min at 37°C. Then 1.5 volumes 96% EtOH and 0.1 volumes sodium acetate 3 M, 5.2 pH were added, and the samples stored at -20°C over night. The DNA was recovered by centrifugation (20 min, 4°C, 15,700 × *g*). The supernatant was carefully removed and the DNA washed with 200 μl of cold 70% EtOH (centrifugation 20 min, 4°C, 15,700 × *g*). The EtOH was removed and the pellet was air dried, before 25 μl TE buffer was added and the DNA re-dissolved at 65°C for 1 h.

A subsample of the isolated DNA was run on a 1% agarose gel stained with GelRed^TM^ (Biotium) and photographed to assess the integrity of the genomic DNA. If the DNA was degraded or contained many short fragments, then paramagnetic beads (Agencourt AMPure XP system, Beckman Coulter Inc.) were used to select for DNA fragments >500 bp. In short, the manufacturers protocol for PCR purification was followed, but fewer beads than suggested were used (volume beads used here = 0.5x DNA volume). The elution buffer was 25 μl TE. Finally, the genomic DNA was quantified with a Qubit® Fluorometer using the dsDNA HS Assay (Invitrogen).

### *SxtA1* and *SxtA4* Sequencing and Cloning

*SxtA1* and *sxtA4* PCRs were run according to Stüken et al. (2011) using primers sxt001/sxt002 and sxt007/sxt008 (**Table 2**), respectively. Selected products were Sanger sequenced using the same primers. *SxtA4* products from strains RCC3227 and RCC3337 were cloned and sequenced using the procedure described in Stüken et al. (2011).

**Table 2:** Primers used in this study.

Name	Use	Sequence (5^′^- 3^′^)	Reference
5.8S-b5^′^ed	5.8S qPCR	GAT GAA GAA TGC AGC AAM ATG	Orr et al. (2013)
5.8S-b3^′^	5.8S qPCR	CAA GCA HAC CTT CAA GMA TAT CC	Galluzzi et al. (2004)
sxt072	*sxtA4* qPCR	CTT GCC CGC CAT ATG TGC TT	Stüken et al. (2013)
sxt073	*sxtA4* qPCR	GCC CGG CGT AGA TGA TGT TG	Stüken et al. (2013)
sxt001	*sxtA1* PCR	TGC AGC GMT GCT ACT CCT ACT AC	Stüken et al. (2011)
sxt002	*sxtA1* PCR	GGT CGT GGT CYA GGA AGG AG	Stüken et al. (2011)
sxt007	*sxtA4* PCR	ATG CTC AAC ATG GGA GTC ATC C	Stüken et al. (2011)
sxt008	*sxtA4* PCR	GGG TCC AGT AGA TGT TGA CGA TG	Stüken et al. (2011)

### Quantitative PCR

All quantitative PCRs (qPCRs) were performed on a Roche LightCycler® 480 system in white 96-well-plates using LightCycler® 480 SYBR Green I Master chemistry (Roche Diagnostics AG, Penzberg, Germany). The sample DNA was freshly diluted with PCR-grade water prior to each experiment. All reactions were run in duplicate or triplicate. Two different qPCRs were run on each DNA, a 5.8S- and a *sxtA4*-targeted qPCR.

The 5.8S qPCR was based on the qPCR assay developed by Galluzzi et al. (2004) but run with a slightly modified protocol as described in Orr et al. (2013). In short, each reaction contained 0.5x SYBR Green I master mix, 150 nM of each primer (5.8S-b5^′^ed and 5.8S-b3^′^, **Table 2**) and between 2, 4, or 8 ng genomic DNA. The cycling protocol was: hot start, one cycle of 95°C for 10 min; amplification, 45 cycles of 95°C for 15 s and 60°C for 45 s, with single acquisition; followed by the melt curve program, one cycle of 95°C for 5 s and 65°C for 1 min, with up to 97°C continuous measurements; and finally, cooling, one cycle of 40°C for 10 s.

The *sxtA* qPCRs were run according to the SYBR Green protocol developed by Stüken et al. (2013). In short, each reaction contained a final concentration of 0.5x SYBR Green I master mix, 125 nM of each primer (sxt072 and sxt073, **Table 2**), and a known quantity of genomic DNA (1, 2, or 4 ng). The cycling protocol was: hot start, one cycle 95°C for 10 min; amplification, 45 cycles 15 s at 95°C, 15 s at 64°C, and 30 s at 72°C; followed by the same melt curve and cooling program as for the 5.8S qPCR (see above). A purified *sxtA4* PCR product generated from genomic DNA of strain CCMP113 with primers sxt007 and sxt008 (**Table 2**) according to Stüken et al. (2011) was run on each *sxtA4* qPCR plate as a standard and contained ∼800,000 *sxtA4* copies.

Quantitative PCR efficiency calculations were based on the kinetics of individual PCR reactions using the algorithm implemented in the *Real-time PCR Miner* (Zhao and Russell, 2005), available online at: http://www.miner.ewindup.info/. To convert the raw data from the Roche LightCycler® 480 system into the right format, the program LC480Conversion.exe originally written for the LinRegPCR program (Ruijter et al., 2009) was used. It is available at: http://www.hartfaalcentrum.nl/.

### *SxtA4* Copy Number Determination

First, the mean efficiency (mean_eff_) of all positive *sxtA4* qPCR reactions and the corresponding slope (*s*) were calculated: $s=\frac{-1}{\log_{10}({mean}_{eff}+1)}$. Then to standardize between the qPCR runs, the intercept (*i*_plate_) for each plate was calculated: $i_{plate}=(\frac{CP1+CP2}{2})-s\times \log_{10}$ STD. CP1 and CP2 were the crossing points of the standard (STD) on each plate. The standard contained ∼800,000 *sxtA4* copies per reaction, see Section “Quantitative PCR” for details. Next, the *sxtA4* CPN in each reaction (CPN_R_) was calculated: ${CPN}_{R}=10\frac{\left({CP}_{R}-i_{plate}\right)}{s}$, where CP_R_ is the crossing point of the individual reaction. Finally, the *sxtA4* CPN per genome (CPN_G_) was estimated by first calculating the *sxtA4* CPN per ng (CPN_ng_):$CPN_{ng}=\frac{CPN_{R}}{DNA_{in}}$, where *DNA*_in_ is the amount of input DNA in ng, and then per genome: ${CPN_{G}=CPN}_{ng}\times \frac{G_{size}}{1000}$, where *G*_size_ is the measured genome size in pg.

### Correlation Analyses

To test for associations between genome size, gene CPN, and total PSTs produced Spearman’s *rho* was calculated using the cor.test function implemented in the R statistical software (package *stats* version 2.14.1).

## Results

### Genome Size Measurements

The *A. minutum* genome sizes are listed in **Table 1** and ranged from 22.5 to 29.6 pg cell^-1^, with an average of 25.7 ± 1.9 pg cell^-1^ (*n* = 15). The genome sizes of toxin group 1 strains (see PST Measurements for toxin profile details) ranged between 22.5 and 29.0 pg and of toxin group 2 strains between 25.4 and 26.9 pg. Strain VGO663 had the biggest genome; a strain in which no PST toxins nor *sxtA4* copies were detected and the sole member of toxin group 3.

### PST Measurements

The results of the toxin analyses are listed in **Tables 1** and **3**. Three groups of PST profiles were observed: (1) GTX1/4 and GTX2/3 (strains isolated from different locations in the Mediterranean Sea and the Atlantic Ocean); (2) C1/2, GTX2/3, and dcGTX2/3 (strains isolated from Brittany, France), and (3) no PST detected (strain VGO663 isolated off Sardinia, Italy).

**Table 3:** PST measurements in fmol cell^-1^.

Strain	Sample	Cells ml^-1^	C1	C2	GTX4	GTX1	dcGTX3	dcGTX2	GTX3	GTX2	Total PST
Al1V	1				0.299	0.143					0.442

VGO722	1	42660			1.776	0.682			0.059	0.029	2.546
	2	17973			2.006	0.208			0.082		2.296
	3	34800			1.802	0.128			0.023	0.011	1.965

AMP13	1	27930			8.038	1.735			0.110	0.034	9.917
	2	23573			6.061	0.755			0.069	0.013	6.897
	3	104733			1.481	0.292			0.009	0.003	1.784

AL10C	1	99030			0.081	0.031					0.112
	2	77653			0.078	0.013					0.091
	3	53200			0.106	0.033			0.003		0.143

VGO942	1	26860			1.220	0.288			0.013	0.005	1.525
	2	14427			0.219						0.219
	3	57667			0.232	0.035			0.033		0.300

AL4V	1				1.777	0.076			0.069	0.025	1.947
	2				2.051	0.217			0.172	0.075	2.516
	3				2.197	0.122			0.217	0.077	2.612

Min3	1	57000			0.564	0.226			0.006		0.797
	2	12880			0.864	0.121			0.008	0.004	0.997
	3	110867			0.123	0.032			0.001	0.001	0.157

VGO577	1	10400			5.632	0.851			0.141	0.021	6.645
	2	23733			9.639	0.915			0.217	0.102	10.874
	3	98400			1.770	0.390			0.026	0.016	2.203

AMP4	1	47800			6.546	3.159			0.351	0.205	10.261
	2	17093			8.731	0.793			0.243	0.044	9.811
	3	38067			4.527	0.654			0.052	0.035	5.267

VGO874	1	40660			0.379	0.200			0.111	0.027	0.717
	2	34293			0.252	0.071			0.034		0.357
	3	36333			0.216	0.290			0.007	0.005	0.517

RCC3227	1	18507			0.049	0.006	0.101	0.016			0.173
	2	6200	0.105	1.893			0.017	0.045	5.513	1.120	8.695
	3	38800	0.219	0.149			0.049	0.006	0.508	0.111	1.043

RCC3337	1	15507			0.007	0.015	1.054				1.076
	2	8853	0.526	1.157			0.014	0.071	1.292	0.058	3.118
	3	75400	0.067	1.062			0.011	0.015	0.081	0.119	1.355

VGO650	1	5600			0.054	0.041	10.337	5.244			15.676
	2	15787	0.848	2.314	0.040	0.054	6.366	3.077			12.698
	3	24467	5.385	2.398	0.043	0.032	6.410	5.103			19.372

VGO651	1	34200			0.083	0.027	6.951	0.973			8.034
	2	53920	4.123	6.247	0.109	0.036	8.605	0.718			19.838
	3	50000	5.406	7.783	0.138	0.046	10.735	1.338			25.446

The total PST content varied considerably between the different strains within each toxin group, and in some cases also between measurements of the same strains. But altogether were the mean and median values higher in toxin group 2 (mean ± SD = 9.710 ± 8.769; median = 8.364; *n* = 12) than in group 1 (mean ± SD = 2.997 ± 3.506; median = 1.865; *n* = 29).

### Quantitative PCR and *sxtA* Gene Copy Numbers Per Genome

The 5.8S qPCR had an amplification efficiency based on all samples of 0.65 ± 0.05 (mean ± SD, *n* = 124). All *Alexandrium* strains amplified with single, specific meltcurve.

**FIGURE 1:**
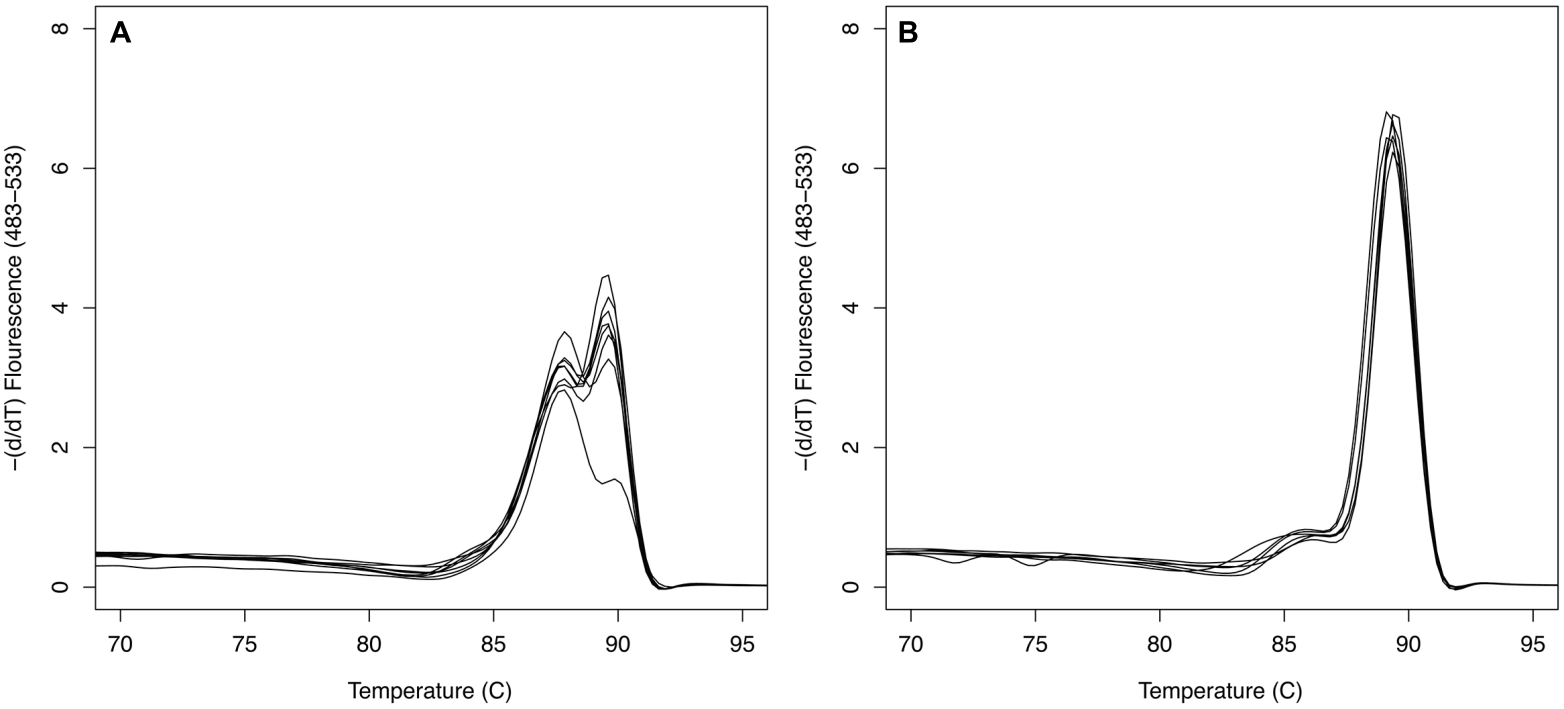
***SxtA4* qPCR melt curve comparison. (A)** Bimodal melting curve of *Alexandrium minutum* strains VGO650, VGO651, RCC3227, and RCC3337 isolated from Brittany, France, producing PST isoforms C1/2, GTX2/3, and dcGTX2/3. **(B)** Single peak melting curves of strains AL1V, VGO577, AL10C, and Min3 producing PST isoforms GTX1/4 and GTX2/3.

The *sxtA* qPCR had an amplification efficiency of 0.94 ± 0.06 (mean ± SD, *n* = 141) and amplification was observed for all *Alexandrium* strains tested, apart from *A. affine* strains CCMP112, *A. andersonii* CCMP2222, and *A. minutum* VGO663. The majority of amplified strains showed a specific melt curve with a single peak. The four *A. minutum* strains isolated from the Channel, Brittany, France (VGO650, VGO651, RCC3337, and RCC3227), however, had a distinct melt curve with a double peak (**Figure 1**). This double peak was due to three SNP loci in the qPCR amplicon only present in the Brittany strains.

The *sxtA4* CPN per genome varied between 1.5 ± 1.0 and 10.8 ± 3.3 (mean ± SD; **Table 1**) in all *A. minutum* strains investigated. The CPNs ranged from 1.5 ± 1.0 to 7.0 ± 5.4 in toxin group 1 and 5.8 ± 2.2 to 10.8 ± 3.3 in toxin group 2 (**Table 1**).

### *SxtA1* and *SxtA4* Amplification and Sequence Diversity

The PCRs for *sxtA1* and *sxtA4* amplified single products of the right size for all PST producing strains. No amplification was observed for *A. minutum* VGO663, as well as the *A. affine* and *A. andersonii* strains.

Direct sequencing of the 670 bp long *sxtA4* fragment resulted in identical sequences without SNPS for strains AL1V, AL10C, AL4, and Min3. The sequences of strain VGO577 and VGO847 had three and six SNPs over the same length. The SNPs were located outside the region used for qPCR and also present in the transcriptome of strain AL1V (Stüken et al., 2011). Strains VGO650 and VGO651, had 14 and 16 SNPs, respectively, none of which coincided with SNPs of the AL1V transcriptome. The *sxtA4* amplicons from RCC3337 and RCC3227 were cloned and sequenced. These sequences confirmed the SNP positions observed in VGO650 and 651. Three of the SNPs observed in VGO650, VGO651, RCC3337, and RCC3227 were located between qPCR primers sxt072 and sxt073 and resulted in two distinct sequences. None of the SNPs observed in any of the *A. minutum* sequences coincided with SNPs in the *A. fundyense* CCMP1719 transcriptome (Stüken et al., 2011), which had 12 SNPs over the same length.

Direct sequencing of the *sxtA1* amplicons (503 bp) resulted in identical sequences for strains AL1V, AL10C, AL4V, Min3, VGO577, and VGO874, sequences from strains VGO650 and VGO651 were 1 bp different.

Sequences have the GenBank accession numbers KM438016–KM438027.

### Correlation Analyses

**FIGURE 2:**
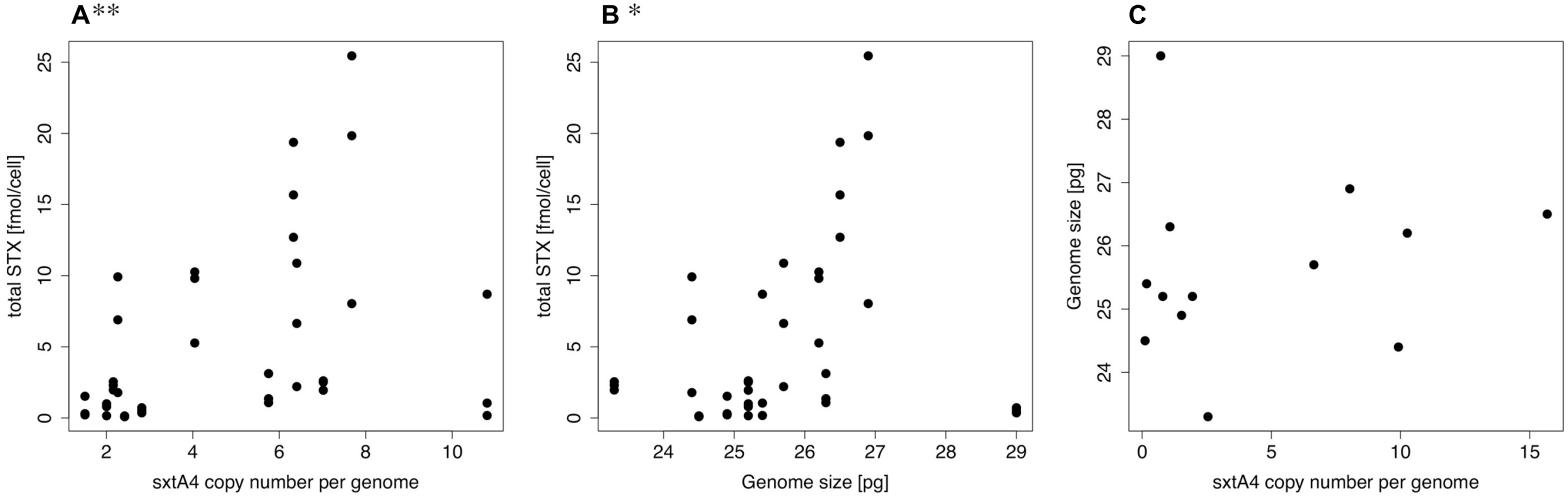
**Spearman rank order correlation plots.** Scatterplots showing the relation between **(A)** *sxtA4* copy number (CPN) per genome and total toxin content per cell, **(B)** total toxin content per cell and genome size, and **(C)** genome size and *sxtA4* CPN per cell. CPN, copy number; significance level: ^∗∗^*p* ≤ 0.01, ^∗^*p* ≤ 0.05.

Only strains for which *sxtA4* could be amplified were included in the analyses. The Spearman rank order correlation results indicated significant positive relations between *sxtA4* CPN per genome and total toxin content per cell (ρ = 0.470, *S* = 4844, *p* = 0.003, **Figure 2A**), and between total toxin content per cell and genome size (ρ = 0.355, *S* = 5896, *p* = 0.029, **Figure 2B**). No significant relation was detected between genome size and *sxtA4* CPN per cell (ρ = 0.473, *S* = 240, *p* = 0.088, **Figure 2C**).

## Discussion

### Relation between *sxtA* Gene Copy Number, PST Content and Genome Size

The results of this study support our previous idea that strains with low levels of PST have fewer copies of the *sxt* genes compared to those with higher toxin levels (Stüken et al., 2011). Specifically, the results show a strong positive correlation between the genomic CPN of *sxtA4* domains and the total cellular content of PST in actively growing *A. minutum* cultures (**Figure 2**). It has been shown that the toxin content and profile of the same *A. minutum* culture throughout the growth curve are generally stable even under different nutrient conditions. Exceptions are limiting conditions, especially the combination of P and N limitations, where toxin content may increase drastically (Flynn et al., 1994). By taking several PST measurements from each strain during active growth and pooling the results, we aimed at capturing the typical variation of toxin production in each strain under standard growth conditions. Thus, our results indicate that strains with a higher genomic *sxtA4* CPN have on average a higher PST content.

Interestingly, the genomic *sxtA4* CPN reported for *A. catenella* strains previously is considerably higher than found for the *A. minutum* strains here. All *A. minutum* strains were estimated to have fewer than 15 copies per genome (**Table 1**), while 100–280 genomic copies have been reported for *A. catenella* (Murray et al., 2011b; Stüken et al., 2011). If *sxtA4* CPN and cell toxin content were also related across species, then the *A. catenella* strains should contain much more toxin compared to the *A. minutum* cells. Apparently, this is not the case; the toxin content per cell reported for three *A. catenella* strains analyzed (Murray et al., 2011b) was comparable or even less than the PST content measured for *A. minutum* here. An alternative explanation for the substantial difference in *sxtA4* CPNs observed in the two species may be linked to their genome sizes. *A. catenella* genomes have been estimated to be two-to-seven times larger than *A. minutum* genomes [64–100 pg; (Hackett et al., 2005; LaJeunesse et al., 2005; Figueroa et al., 2010)]. The reason for this size difference is not clear, but may be related to ploidy differences. In this case, *sxtA4* CPN would not be a positively selected trait but rather a result of genome dynamics.

However, within the species *A. minutum sxtA4* CPN did not scale with genome size (**Figure 2**), even though we observed considerable differences in genome size (**Table 1**). Thus, *sxtA4* CPN does not appear to be a function of genome size in *A. minutum*. This is the first time that so many strains of the same dinoflagellate species have been measured and that such a breath of genome size within a species has been documented. It is currently unclear what causes the genome size diversity but it might be related to chromosomal differences, as aneuploidy may be common in dinoflagellate cultures (Loper et al., 1980).

We also observed a positive correlation between genome size and toxin content (**Figure 2**; **Table 3**). It is unclear what caused this relationship, as toxin content and *sxtA4* CPN were positively correlated, but *sxtA4* CPN and genome size were not. In dinoflagellates DNA content correlates with cell size (LaJeunesse et al., 2005; Dapena et al., 2015) a relation that has been document for most eukaryotic lineages, e.g., (Gregory, 2001; Cavalier-Smith, 2005). It is possible that cells with a higher cell volume contain more PSTs. However, studies comparing toxin content of different sized cells have, to our knowledge, not been undertaken yet.

### Non-PST Producing *A. minutum* Strain VGO663

For one of the *A. minutum* strains, strain VGO663, neither PST nor *sxtA1*, or *sxtA4* gene fragments were detected. While it was previously known that the species *A. minutum* contains PST-producing and non-producing strains, e.g., (Touzet et al., 2007), this is the first *A. minutum* strain for which no *sxt* genes could be amplified. The finding weakens our previous hypothesis that all strains of PST-producing *Alexandrium* species contain *sxtA1* and *sxtA4* (Stüken et al., 2011). It also adds to the growing body of evidence that the presence of *sxtA1* and *sxtA4* is not only a pre-requisite for PST synthesis, but is also a good indicator for its actual synthesis. A recent study of PST-producing and non-producing *A. ostenfeldii* strains showed that all PST-producing strains contained both, *sxtA1* and *sxtA4*, while either *sxtA4* or *sxtA1* and *sxtA4* were not detected in non-PST-producing strains (Suikkanen et al., 2013). Also, while all *A. tamarense* strains investigated so far contained both *sxtA1* and *sxtA4*, it has been indicated that some strains classified not to produce PSTs, might indeed produce low amounts or unusual isoforms (Negri et al., 2003; Orr et al., 2011; Murray et al., 2012).

### Toxin Profiles and *sxtA* Gene Diversity

Three groups with different toxin profiles were observed in the 15 *A. minutum* strains analyzed. The first group of strains isolated from different locations in the Mediterranean Sea and the Atlantic Ocean produced pre-dominantly GTX4 as well as smaller amounts of GTX1-3, the second group isolated from Brittany, France, produced mainly GTX3, but also substantial amounts of GTX2, C2, and C1, and smaller amounts of dcGTX2 and dcGTX3. The third group, strain VGO663, did not produce any measurable amounts of toxin (**Table 3**).

There was no discernable difference in total PST produced, genome size or number of *sxtA4* copies per genome between *A. minutum* toxin group 1 and 2. There was, however, a marked difference in the diversity of *sxtA* gene copies between the two groups. This difference was clearly visible in the melt curve analyses of the *sxtA* qPCR amplicon: all strains of the first group had a characteristic single peak melt curve, while the strains from the second group displayed a bimodal curve (**Figure 1**). Detailed analyses showed that this melt curve pattern was due to the presence of three SNP sites resulting into two different *sxtA* gene sequences in the strains from Brittany over the lengths of the qPCR amplicon. None of the other *A. minutum* strains contained SNPs in this region. Further, analyses of the longer *sxtA4* PCR amplicon generated with primers sxt007 and sxt008 showed a much higher sequence variation in the *A. minutum* strains isolated from Brittany compared to the rest of the *A. minutum* strains analyzed. The sequence variation between these two groups seems to have developed independently – none of the SNPs coincided.

It is not clear, if the sequence variations observed are related to the PST profile produced or the site of isolation. The first scenario is unlikely because it is probably not *sxtA* that determines which isoforms of PST are produced, but rather the presence and functioning of tailoring genes, as has been shown for cyanobacteria (Kellmann et al., 2008; Soto-Liebe et al., 2010; Murray et al., 2011a). As the entire PST pathway in dinoflagellates has not been characterized, this is currently difficult to investigate. The second scenario is more plausible. It indicates the presence of an *A. minutum* population at the coast of Brittany in which the *sxtA* genes evolve independently from the rest of *A. minutum* strains in the region. Microsatellite analyses support the presence of distinct *A. minutum* populations in the Mediterranean Sea (McCauley et al., 2009; Casabianca et al., 2012). If this is true, then it implies that *sxtA* evolves not only in different species as has been suggested earlier (Stüken et al., 2011) but also in different entities within a species. In this context it is also interesting to note that the high sequence variation was only observed in domain *sxtA4*, not in *sxtA1*. It is possible that these two domains are under different selection pressures. This has already been indicated by the finding that only *sxtA1* could be amplified in some *A. ostenfeldii* strains (Suikkanen et al., 2013). Further, it might give an explanation why it was so far not possible to amplify the entire *sxtA* gene from dinoflagellate gDNA – the two domains may be encoded in different regions of the genome and are spliced together with domains *sxtA2* and *sxtA3* to form entire *sxtA* transcripts.

## Conclusion and Future Perspectives

The cellular regulation of PST-synthesis in dinoflagellates remains complex. Here, we have observed a strong positive relation between genomic *sxtA4* CPN and PST content in actively growing *A. minutum* cells. This indicates that the number of *sxt* gene copies per genome may determine the amount of STX that is produced by dinoflagellate cells. It is not clear, however, if the CPN determines the maximum amount of toxin that can be produced, or if it is just an indicator for the amount of toxin that is produced under unconstraint growth conditions. For example, it has been shown that growth rate and PST synthesis in *Alexandrium* species are related during logarithmic growth, but that this association weakens as nutrients are depleted and the cells enter stationary phase (Boczar et al., 1988; Anderson et al., 1990; Parkhill and Cembella, 1999). Depending on the nutrient limitation, either more or less toxin is produced per cell (Anderson et al., 1990). Thus, it would be informative to investigate if strains with more CPNs per genome generally produce more toxins during unconstraint growth than those with less CPNs. Indeed, preliminary results of this group indicate that *A. minutum* cells with a higher *sxtA4* CPN per genome have a higher toxin content during exponential growth phase than those with less CPNs, and that this relation lessens as nutrients are depleted and cell growth slows down. However, further experiments are needed to verify such a conclusion. In addition, it would be insightful to test if strains with more CPNs have a higher phenotypic plasticity regarding toxin production, i.e., if their response to extreme conditions shows a higher variation in toxin production than strains with fewer CPNs. Nevertheless, it is so far still unclear if genomic *sxt* gene CPN is indeed the bottleneck in PST synthesis. To investigate this, parallel analyses including toxin synthesis, genomic CPNs, and transcript abundance of a range of strains are needed. Wiese et al. (2014) have recently published a *sxtA4* qPCR assay that includes normalization targets and will facilitate such an analysis.

Our results also show that *sxtA* sequence variability differs between *A. minutum* strains. For *A. catenella* it has recently been shown that preferential transcription of certain gene copies and mRNA editing may play important roles in the maturation of *sxtA* transcripts (Wiese et al., 2014). It will be interesting to investigate, if the same is also true for *A. minutum* and if this may have an impact on the total amount of toxin produced.

Finally it remains to be established if the same mechanisms regulate PST-synthesis in all *Alexandrium* species. Recent analyses of the ribosomal DNA in various *Alexandrium* species revealed distinct chromosomal organization (Figueroa et al., 2014).

## Conflict of Interest Statement

The authors declare that the research was conducted in the absence of any commercial or financial relationships that could be construed as a potential conflict of interest.
